# Complex Thinking as a Predictor of Anxiety Symptoms in Medical Students

**DOI:** 10.1007/s40670-026-02863-9

**Published:** 2026-09-05

**Authors:** Sebastián Soto-García, Ofelia Gómez-Landeros, Efrén Raúl Ponce-Rosas, Anna Vitalievna Sokolova-Grinovievkaya

**Affiliations:** 1 Department of Health Care, CBS Division, Autonomous Metropolitan University, Los Colorines, Mexico; 2 Family Medicine Subdivision, Division of Graduate Studies, School of Medicine UNAM, Ciudad de México, Mexico; 3 Department of Policy and Culture, CSH Division, Autonomous Metropolitan University, Los Colorines, Mexico

**Keywords:** Complex thinking, Anxiety symptoms, Medical students, Predictive model, COMPLEX-21, Beck Anxiety Inventory

## Abstract

**Introduction:**

Medical training is associated with a high prevalence of anxiety symptoms that escalate with curricular advancement. Complex thinking has been proposed as a protective cognitive skill; however, its predictive capacity and evolution throughout training remain poorly explored. This study aimed to determine the predictive capacity of complex thinking for anxiety symptoms in medical students and to analyze the variation of this predictive effect by academic year.

**Methods:**

A cross-sectional study was conducted among 567 medical students at a Mexican public university. Complex thinking and anxiety symptoms were assessed using the COMPLEX-21 scale and the Beck Anxiety Inventory, respectively. One-way ANOVA with Tukey post hoc comparisons, Pearson correlations, stratified linear regression models, and a Year × Complex Thinking interaction model were conducted.

**Results:**

In the overall sample, complex thinking was an inverse predictor of anxiety symptoms (*B* = − 0.249, 95% CI, − 0.33 to − 0.17; *R*^*2*^ = 0.062; *p* < .001). Mean anxiety scores were higher in the third year than in the first year (*M* = 21.52 vs. *M* = 17.57; Tukey-adjusted *p* = .039), whereas complex-thinking scores remained stable across academic-year groups (range, 81.14–84.14; ANOVA *p* = .233). Stratified models showed descriptively higher explained variance in the third (*R*^*2*^ = 0.110) and fourth (*R*^*2*^ = 0.098) years than in the first year (*R*^*2*^ = 0.026). However, the Year × Complex Thinking interaction was not statistically significant (*p* = .058).

**Conclusions:**

Complex thinking showed a consistent inverse predictive relationship with anxiety symptoms across academic-year groups. The stability of its levels suggests a potential curricular gap that warrants further evaluation. Integrating complex reasoning into pedagogical models may support student well-being and educational quality.

## Introduction

The academic journey in medicine is a highly demanding period, characterized by intense cognitive load and early exposure to burnout [1–4]. Previous studies suggest that medical students may experience a higher prevalence of anxiety and depressive symptoms than students in other fields, with particularly high levels reported during later stages of training [5]. In the Mexican context, this disparity is alarming: it has been documented that medical students are up to 32 times more likely to exhibit symptoms of anxiety compared to students in other fields, with a prevalence ranging from 30.4% in the first year to 94.4% in the final years [5]. Stressors such as long work hours, sleep deprivation, financial pressures, and the inherent demands of the medical profession contribute significantly to the onset of depression and anxiety in medical students [6–9].

Consequently, this environment of constant pressure predisposes students to anxiety and increases the risk of developing other mental health problems, such as academic burnout syndrome [10, 11]. This phenomenon, closely linked to chronic stress [7, 8, 12], underscores the need to identify factors that mediate students’ emotional responses to the academic, clinical, and social challenges of their education.

Given the multidimensional nature of these challenges, complex thinking stands as a relevant theoretical framework. As conceptualized by Edgar Morin, it proposes a reform of knowledge that allows for the articulation of fragmented bodies of knowledge. In his work *The Seven Complex Lessons in Education for the Future*, the author argues that education must prepare students to face the uncertainty inherent in human existence and to integrate knowledge that a disciplinary approach tends to fragment [13]. The incorporation of complex thinking into university education is considered essential for fostering a comprehensive understanding of disciplinary phenomena through the integration of multiple interconnected dimensions of knowledge rather than their isolated analysis [13, 14]. Medical education provides a particularly appropriate context for applying this framework because physicians must simultaneously integrate biomedical knowledge, clinical reasoning, communication skills, ethical decision-making, and psychosocial factors while making decisions under conditions of uncertainty [15].

Unlike the graduate-entry medical programs that predominate in countries such as the United States and Canada, medical education in Mexico is generally offered as a direct-entry undergraduate degree following completion of high school [16]. At the institution where this study was conducted, the Bachelor of Medicine is a direct-entry undergraduate program with a planned duration of 12 academic trimesters and one year of undergraduate medical internship. It follows an integrated modular curriculum, in which basic biomedical sciences, clinical sciences, and social and community health are articulated around specific health problems rather than being taught as isolated disciplines. The curriculum includes early community and clinical experiences, an undergraduate medical internship, followed by a mandatory social service year, promoting the progressive development of competencies required for medical practice. This integrated, competency-oriented educational model makes the development of complex thinking especially relevant for understanding and addressing multifactorial health problems.

In the field of medical education, complex thinking serves as a conceptual framework that enables future physicians to adopt a systemic perspective on the health–disease continuum, thereby facilitating decision-making under the uncertainty inherent in clinical practice [17]. This need is corroborated by recent evidence that positions clinical competence not as a mere technical skill, but as an intrinsically complex construct that requires the deployment of critical and scientific thinking to manage patient dynamics [18]. In this regard, Lifshitz and Pomposo [19] propose that complex thinking should be understood not only as theoretical content but also as a fundamental pedagogical strategy for recognizing the multidimensionality of the human being.

Although resilience and critical thinking are also recognized as important competencies in medical education, they represent conceptually distinct constructs. Resilience refers to the capacity to adapt positively to adversity and recover from stressful experiences [20], whereas critical thinking is primarily concerned with the purposeful analysis and evaluation of information to support reasoned judgments [21]. In contrast, complex thinking encompasses systemic reasoning, multidimensional analysis, metacognition, creativity, ethical reflection, and the ability to integrate diverse sources of knowledge while managing uncertainty [22]. Consequently, resilience and critical thinking may be regarded as complementary dimensions within the broader framework of complex thinking rather than alternative explanatory constructs, thereby positioning complex thinking as the central predictive construct examined in the present study.

When addressing the psychological demands of medical education, emotional skills such as resilience have often been proposed as critical determinants of students’ well-being [23, 24]. However, as established above, resilience primarily reflects adaptive responses to adversity, whereas complex thinking encompasses the cognitive and metacognitive processes through which students integrate knowledge, interpret uncertainty, and respond to complex academic and clinical situations. From this perspective, complex thinking emerges as a comprehensive construct that facilitates the management of multidimensional phenomena [25]. By integrating dimensions such as systemic analysis, metacognition, creativity, and problem-solving, this competency enables students to understand the multifactorial nature of medical practice and address challenges in conditions of uncertainty [25]. Likewise, metacognitive processes and critical analysis promote reflection on one’s own performance, which, when combined with creativity to propose alternative solutions, constitutes a regulatory mechanism that could mitigate anxiety symptoms by providing students with a greater sense of cognitive control and agency [25].

Despite the theoretical relevance of these constructs, empirical evidence directly examining the relationship between complex thinking and anxiety symptoms among medical students remains limited, particularly regarding whether this relationship varies across the different stages of medical education, which are characterized by distinct academic and clinical demands.

Given the above, the primary objective of this study is to determine the predictive capacity of complex thinking for anxiety symptoms in medical students enrolled in different years of a competency-based Bachelor of Medicine program. More specifically, the study seeks to determine whether the strength of this predictive relationship differs across the academic years of medical training.

## Methods

### Research Design and Context

An observational, cross-sectional, descriptive, and comparative study was conducted in accordance with the STROBE guidelines [26] at a public university in Mexico in 2026. The medical curriculum at this institution is organized into 12 trimesters, ranging from basic sciences to clinical rotations. For statistical and comparative analysis, the student population was stratified into four academic levels based on their curricular progress: First Year (Trimesters 1–3), Second Year (Trimesters 4–6), Third Year (Trimesters 7–9), and Fourth Year (Trimesters 10–12).

## Participants and Procedure

A nonprobability purposive sampling strategy was used, with the aim of recruiting all active students. The inclusion criteria were: (1) being enrolled in any of the 12 trimesters of the Bachelor of Medicine program, (2) agreeing to participate voluntarily, and (3) completing the entire survey process.

Data collection was conducted using a self-administered electronic questionnaire designed in Google Forms. To standardize the administration conditions and maximize the response rate, groups were convened per trimester cohort at the institution’s computer labs, where they completed the questionnaire simultaneously during class time.

Due to the large student population, data collection was carried out over two consecutive weeks (weeks 4 and 5 of the trimester) for all academic cohorts. This specific timeframe—corresponding to the first third of the term—was selected because it reflects a period of standard academic workload. Surveying students during these weeks avoided both the acute stress spikes caused by end-of-trimester evaluations and the atypically low workload associated with the start of the term. Because the BAI assesses symptoms experienced during the previous week, participants were instructed to report symptoms experienced during the seven days immediately preceding questionnaire completion.

Of a total eligible population of 757 students enrolled at the time of the study, 611 (80.7%) agreed to participate. Following a data cleaning phase, 42 records were excluded due to incomplete questionnaires, and two outliers were identified using box plots (values greater than 1.5 times the interquartile range). The final analytical sample consisted of 567 students (effective response rate of 74.9%).

### Statistical Analysis

Data analysis was performed using IBM SPSS Statistics, version 26.0. For the predictive models, the independent variable (complex thinking) and the dependent variable (anxiety symptoms) were operationalized and analyzed as continuous variables based on their total scores. This continuous approach was selected to preserve the full variance of the data and maximize the statistical power of the predictive analysis. These continuous variables were described using means and standard deviations, whereas categorical variables (such as gender and academic year) were described using frequencies and percentages.

Prior to performing inferential statistics, the parametric assumptions of the multivariate distribution of anxiety and complex thinking scores were evaluated. For this purpose, Kline’s criterion [27] was used, first calculating Mahalanobis distances and comparing the results with the number of variables included in the model. Kline’s reference value was 13.815, which was not exceeded by any case in the study sample. It was concluded that the multivariate distribution satisfied the assumption of normality, thus justifying the use of linear regression models. Additionally, to evaluate the differences in these variables by academic year, a one-way analysis of variance (ANOVA) was used, followed by Tukey’s post hoc test for multiple comparisons.

Furthermore, to evaluate the specific associations between the individual domains of complex thinking and anxiety symptoms without introducing multicollinearity into the predictive models, a bivariate Pearson correlation analysis was conducted, stratifying the data by academic year.

Finally, to formally evaluate whether the predictive capacity of complex thinking on anxiety varied significantly across academic years, a moderated multiple regression analysis was conducted, including an interaction term (Year × Complex Thinking).

## Instruments and Measures

### Complex Thinking (COMPLEX-21 Scale)

To assess complex thinking, defined as the independent variable in this study, the COMPLEX-21 scale developed by Tobon and Luna-Nemecio [25] was used. This instrument was selected over alternative instruments (such as the Watson-Glaser or Cornell critical thinking tests) due to its capacity to assess complex thinking as an integrated, non-fragmented construct. It comprises 21 items evaluated on a five-point Likert-type scale (1 = *never* to 5 = *always*). The scale encompasses five dimensions: (1) problem-solving (5 items), defined as the capacity to engage in cognitive processing to understand and resolve problem situations where a method of solution is not immediately obvious; (2) critical analysis (5 items), an intellectual process that involves actively and skillfully conceptualizing, applying, analyzing, synthesizing, or evaluating information as a guide to trust and action; (3) metacognition (4 items), which refers to the knowledge concerning one’s own cognitive processes, including the active monitoring and consequent regulation of these processes in the service of a concrete goal; (4) systemic analysis (3 items), a methodology for analyzing and solving problems using systemic research and the comparison of alternatives; and (5) creativity (4 items), a divergent thinking process consisting of the competence to provide alternative answers based on the information provided. Consequently, the possible score ranges vary for each specific dimension based on its number of items: problem-solving and critical analysis range from 5 to 25 points each; metacognition and creativity range from 4 to 20 points each; and systemic analysis ranges from 3 to 15 points. Higher scores indicate a greater frequency of application of that specific complex thinking skill.

The original validation of the instrument was conducted using confirmatory factor analysis with the maximum likelihood estimation method. That study demonstrated adequate convergent validity, evidenced by Average Variance Extracted (AVE) values above the 0.50 threshold for all factors, as well as strict discriminant validity verified via the Fornell-Larcker criterion (where the square root of the AVE for each factor was consistently greater than its correlations with the other dimensions). It also reported robust goodness-of-fit indices (CFI = 0.971, TLI = 0.963, RMSEA = 0.045) and composite reliability coefficients ranging between 0.794 and 0.867 [25].

### Anxiety Symptoms (Beck Anxiety Inventory, BAI)

To measure anxiety symptoms as the dependent variable, the Beck Anxiety Inventory (BAI) was administered [28]. The instrument consists of 21 items rated on a four-point Likert-type scale (0 *= not at all* to 3 = *severely*), focusing on somatic and cognitive symptoms experienced during the past week. Total scores allow anxiety to be classified into four levels: minimal (0–7), mild (8–15), moderate (16–25), and severe (26–63) [29]. The BAI is internationally recognized for its high convergent validity and discriminant ability relative to other affective disorders [30]. Additionally, recent evidence has confirmed its cross-cultural validity, demonstrating minimal measurement bias and high clinical equivalence in Hispanic populations and in Spanish-language administrations, which supports its suitability for the study sample [31].

## Ethical Considerations

The study protocol was approved by the Research Ethics Committee of the Division of Biological and Health Sciences at Universidad Autónoma Metropolitana, Xochimilco campus (Reference No. CEI.2026.009). All procedures were conducted in accordance with the ethical principles set forth in the World Medical Association’s Declaration of Helsinki (revised in 2024) [32], the UNESCO Universal Declaration on Bioethics and Human Rights [33], the Regulations of Mexico’s General Health Law on Health Research [34], the provisions of the Code of Ethics of the American Educational Research Association (AERA) [35], and the Code of Fair Practice in Educational Assessment [36]. Prior to participation, all students were informed of the research objectives and voluntarily provided digital informed consent. All data were strictly anonymized, ensuring that no participant could be identified by the research team. Additionally, a universal safety protocol was implemented, which involved providing all participants with an institutional and national directory of specialized psychological support resources.

## Results

### Sociodemographic Characteristics of the Participants

The final analytical sample consisted of 567 medical students. As shown in Table 1, the majority of the participants were female (75.3%), and the overall mean age was 20.45 years (SD = 1.81, range = 17–30). The sample was relatively evenly distributed across the four academic years, with a progressive and expected increase in mean age corresponding to curricular advancement.

**Table 1:** Sociodemographic characteristics of the medical student sample

Characteristic	Overall Sample (*N* = 567)	1st Year (*n* = 150)	2nd Year (*n* = 162)	3rd Year (*n* = 147)	4th Year (*n* = 108)
Age, years					
Mean (SD)	20.45 (1.81)	19.09 (1.23)	19.86 (1.38)	21.03 (1.21)	22.44 (1.69)
Age range (years)	17–30	17–25	18–26	19–25	20–30
Gender, n (%)					
Female	427 (75.3%)	112 (74.7%)	114 (70.4%)	121 (82.3%)	80 (74.1%)
Male	140 (24.7%)	38 (25.3%)	48 (29.6%)	26 (17.7%)	28 (25.9%)

Total sample size (*N**) = 567;**n**= *subsample size. Data are presented as means (Standard Deviation, SD) and ranges for continuous variables, and as frequencies (percentages) for categorical variables

### Internal Consistency and Reliability of the Instruments

The reliability of the scales was assessed using Cronbach’s alpha, demonstrating excellent internal consistency in the study sample (*N* = 567). The Complex Thinking Scale (COMPLEX-21) yielded an α of 0.946, while the Beck Anxiety Inventory (BAI) obtained an α of 0.936. These coefficients confirmed the psychometric robustness of both instruments for the evaluated population.

### Comparative Analysis of Variables by Academic Year

Prior to predictive modeling, the descriptive analysis (Table 2) revealed an upward trend in mean anxiety scores from the first year (*M* = 17.57) until reaching a peak in the third year (*M* = 21.52). The pattern of anxiety symptoms throughout academic training was explored using a one-way ANOVA (Table 3), yielding a small effect size (*η*_*p*_^*2*^ = 0.014). Tukey’s post hoc tests confirmed that this difference between the first and third years was statistically significant (*p* = .039). Although the fourth year maintained high levels (*M* = 20.77), the difference compared to the first year did not reach statistical significance (*p* = .192), despite the magnitude of the observed mean. These data suggest that anxiety levels increased notably midway through the medical program.

Similarly, we assessed whether there were differences in the level of complex thinking according to the year of training. The ANOVA (Table 3) indicated that there were no statistically significant changes in this competency throughout the program, with a negligible effect size (*η*_*p*_^*2*^ = 0.008) and means remaining stable across academic years (Table 2; *M* range: 81.14–84.14). This demonstrated that, within this sample, mean complex-thinking scores remained statistically stable across academic-year groups.

Furthermore, to provide a granular and comprehensive overview of this construct, aggregated summary statistics for each specific domain of the COMPLEX-21 scale (problem-solving, critical analysis, metacognition, systemic analysis, and creativity) were calculated. These domain-level distributions are detailed in Table 2, presenting both the overall sample means and the descriptive breakdown by academic year. As detailed in the Instruments section, it is important to note that the absolute mean scores vary across dimensions due to the differing number of items in each subscale (ranging from 3 to 5 items), which determines their respective maximum possible scores. Consistent with the global complex thinking score, the descriptive data indicate that the mean scores of all individual domains remained remarkably stable across the four academic years.

**Table 2:** Descriptive statistics of anxiety symptoms and complex thinking scores by academic year

Variable	Overall M (SD)	1st Year M (SD)	2nd Year M (SD)	3rd Year M (SD)	4th Year M (SD)
Anxiety Symptoms	19.81 (12.77)	17.57 (11.54)	19.71 (12.49)	21.52 (13.03)	20.77 (14.09)
Complex Thinking	82.83 (12.82)	83.33 (12.19)	81.14 (13.18)	83.20 (12.75)	84.14 (13.17)
Problem solving	20.35 (3.55)	20.39 (3.41)	19.95 (3.64)	20.52 (3.49)	20.65 (3.67)
Critical analysis	19.87 (3.49)	20.02 (3.51)	19.49 (3.46)	19.97 (3.48)	20.08 (3.54)
Metacognition	16.03 (2.87)	16.05 (2.73)	15.76 (2.89)	16.18 (2.87)	16.22 (3.03)
Systemic analysis	11.28 (2.33)	11.35 (2.15)	10.97 (2.45)	11.23 (2.29)	11.73 (2.37)
Creativity	15.29 (2.85)	15.51 (2.84)	14.98 (2.97)	15.29 (2.76)	15.45 (2.81)

Data are presented as Mean (*M*) and Standard Deviation (*SD*). Total sample size was *N* = 567

**Table 3:** One-way analysis of variance (ANOVA) comparing anxiety symptoms and complex thinking across academic years

Variable	df_between_	df_within_	F	*p*	η_*p*_^2^
Anxiety Symptoms	3	563	2.64	0.049*	0.014
Complex Thinking	3	563	1.43	0.233	0.008

Total sample size (*N*) = 567. Statistical analysis was performed using one-way ANOVA. *df* = degrees of freedom; *η*_*p*_^*2*^ = partial eta squared (effect size). * *p*< .05

### Correlational Analysis Between Complex Thinking Domains and Anxiety

To further examine the associations between specific cognitive domains and anxiety symptoms, bivariate Pearson correlations were calculated between the five domains of the COMPLEX-21 scale and BAI total scores, stratified by academic year (Table 4). In the overall sample, all complex thinking domains exhibited a significant inverse relationship with anxiety. Notably, the domain-level analysis revealed shifting dynamics across the academic progression. During the first and second years, systemic-analysis and creativity scores showed statistically significant inverse correlations with BAI scores. In the third- and fourth-year groups—typically characterized by higher clinical exposure and academic demands—problem-solving showed the largest-magnitude inverse correlation with BAI scores (*r* = − .402, *p* < .01 in the third-year group). Other domains also showed inverse correlations of varying magnitude across the academic-year groups.

**Table 4:** Pearson correlation matrix between complex thinking domains and anxiety (BAI) by academic year

Variable	Overall Sample (*N* = 567)	1st Year (*n* = 150)	2nd Year (*n* = 162)	3rd Year (*n* = 147)	4th Year (*n* = 108)
Problem solving	− 0.247**	− 0.126	− 0.187*	− 0.402**	− 0.294**
Critical analysis	− 0.159**	− 0.036	− 0.092	− 0.242**	− 0.291**
Metacognition	− 0.209**	− 0.174*	− 0.189*	− 0.245**	− 0.249**
Systemic analysis	− 0.226**	− 0.221**	− 0.213**	− 0.274**	− 0.210*
Creativity	− 0.227**	− 0.162*	− 0.241**	− 0.238**	− 0.272**

Values are Pearson correlation coefficients between each COMPLEX-21 domain and the total BAI score. All correlations were two-tailed. *p < .05 (two-tailed). ** *p* < .01 (two-tailed)

### Predictive Relationship Between Complex Thinking and Anxiety Symptoms

A simple linear regression model was used to estimate the capacity of complex thinking to predict anxiety symptoms (Table 5). Complex thinking served as the predictor variable and anxiety symptoms as the outcome. In the overall sample (*N* = 567), the model was statistically significant (*F*(1, 565) = 37.63, *p* < .001), explaining 6.2% of the variance in anxiety (*R²* = 0.062). The unstandardized coefficient revealed a significant inverse relationship (*B* = -0.249, *SE* = 0.041, β = − 0.250, 95% CI [-0.33, -0.17]), indicating that higher levels of complex thinking predicted lower anxiety scores.

When analyzing the model by academic year, variations in the model’s predictive power were observed. In the first-year group (*n* = 150), the model reached borderline significance (*F*(1, 148) = 3.96, *p* = .048), explaining only 2.6% of the variance (*R²* = 0.026). The upper limit of the confidence interval included zero (*B* = -0.153, *SE* = 0.077, β = − 0.161, 95% CI [-0.31, 0.00]), indicating that, at this initial stage, there was insufficient statistical evidence to affirm a robust relationship between complex thinking and anxiety symptoms.

In contrast, starting in the second academic year (*n* = 162), the model reached statistical significance (*F*(1, 160) = 7.44, *p* = .007), with an inverse coefficient indicating that higher complex-thinking scores predicted lower anxiety symptoms (*B* = -0.200, *SE* = 0.073, β = − 0.211, 95% CI [-0.34, -0.06]) and an explained variance of 4.4% (*R²* = 0.044).

**Fig. 1:**
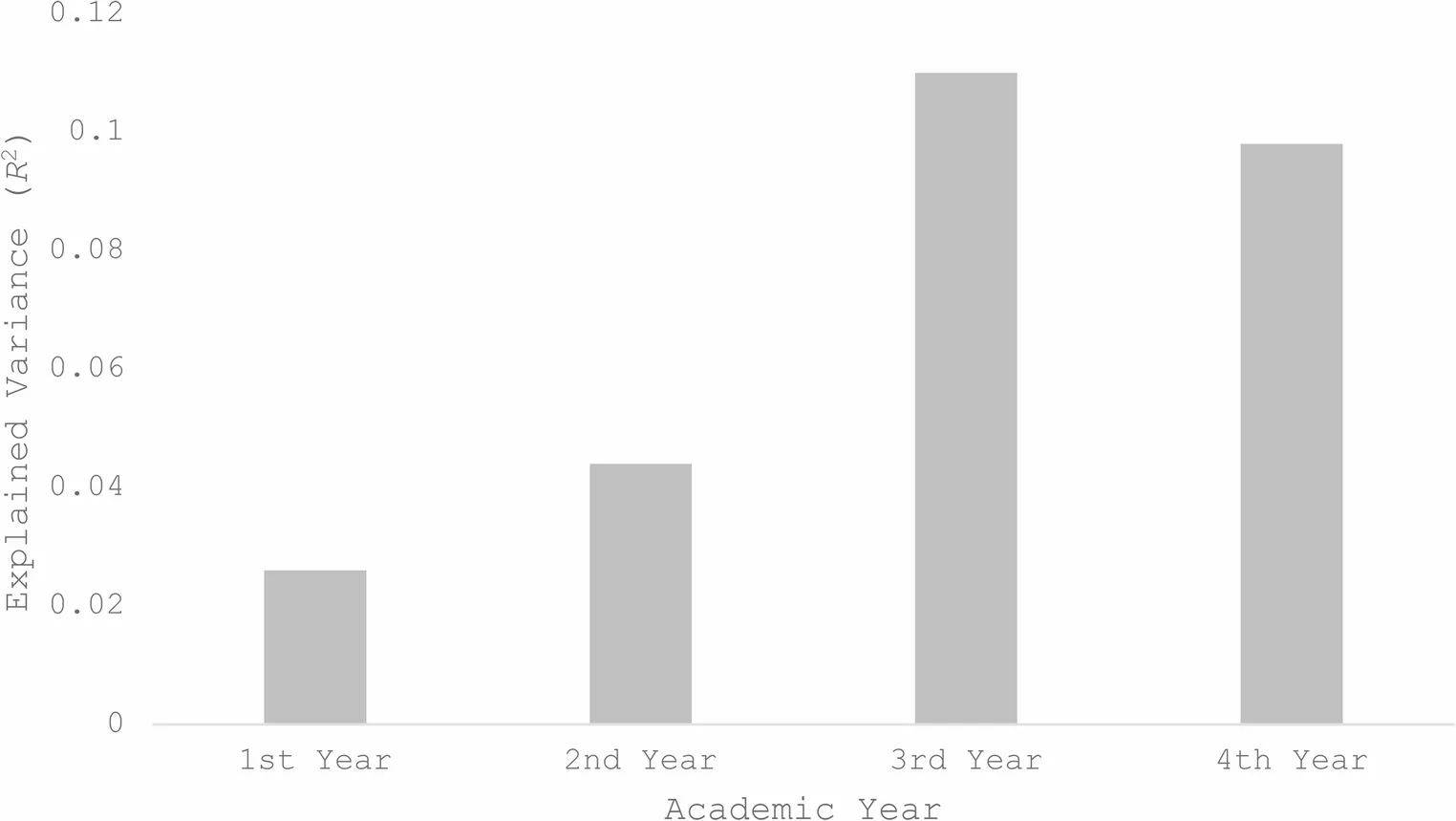
Trend of explained variance. The bar chart displays the proportion of variance in BAI scores explained by complex thinking across academic-year groups. The *R*^*2*^ values were descriptively higher in the third- and fourth-year groups than in the first-year group

Descriptively, higher values of explained variance were observed in the upper grades (Fig. 1). In the third year (*n* = 147), the model explained 11% of the variance (*R²* = 0.110), demonstrating a statistically significant inverse relationship (*F*(1, 145) = 17.99, *p* < .001; *B* = -0.340, *SE* = 0.080, β = − 0.332, 95% CI [-0.50, -0.18]).

Finally, in the fourth year (*n* = 108), the model explained 9.8% of the variance in BAI scores (*R²* = 0.098), with statistical significance (*F*(1, 106) = 11.53, *p* = .001). In this group, higher levels of complex thinking predicted lower anxiety symptom levels (*B* = -0.335, *SE* = 0.099, β = − 0.313, 95% CI [-0.53, -0.14]).

To formally test whether the protective effect of complex thinking strengthened across academic years, a moderated multiple regression was conducted including an interaction term (Year × Complex Thinking). The interaction model did not reach statistical significance (*p* = .058), indicating that the analysis did not provide statistical evidence that the predictive relationship differed across academic-year groups.

**Table 5:** Simple linear regression models to predict anxiety symptoms based on complex thinking, by academic year

Year	*N*	*R* ^2^	F	Intercept (β_0_)	B	SE	β (Standardized)	*p*	95% CI
Overall Sample	567	0.062	37.63	40.421	-0.249	0.041	− 0.250	< 0.001	[-0.33, -0.17]
1st year	150	0.026	3.96	30.310	-0.153	0.077	− 0.161	0.048	[-0.31, 0.00]
2nd year	162	0.044	7.44	35.940	-0.200	0.073	− 0.211	0.007	[-0.34, -0.06]
3rd year	147	0.110	17.99	49.780	-0.340	0.080	− 0.332	< 0.001	[-0.50, -0.18]
4th year	108	0.098	11.53	48.960	-0.335	0.099	− 0.313	0.001	[-0.53, -0.14]

*B* = unstandardized regression coefficient; *SE* = standard error; β = standardized regression coefficient; *CI* = confidence interval. The dependent variable is anxiety symptoms (BAI total score)

## Discussion

The findings of the present study revealed an inverse and statistically significant predictive relationship between complex thinking and anxiety symptoms in the general sample. This result suggests that this cognitive competence provides a relevant explanatory capacity regarding the emotional burden associated with medical training, acting as a protective factor. This empirical finding is consistent with the theoretical proposals of Morin [13] and Lifshitz [14], who argue that the incorporation of complex thinking into the university setting not only improves educational quality but also equips students with tools to cope with the uncertainty inherent in their discipline. Likewise, it aligns with recent research identifying higher-order cognitive resources and resilience as key elements for mitigating academic burnout in health sciences students [10].

Furthermore, while the stratified analysis showed descriptive variations in the explained variance across academic years (from 2.6% in the first year to 11% in the third year and 9.8% in the fourth year), the formal interaction test indicated that these differences did not reach statistical significance. Therefore, rather than a progressive strengthening, the data suggest that complex thinking acts as a consistent protective factor regardless of the specific educational stage.

To understand this phenomenon, it is necessary to analyze the individual behavior of the constructs over time. On the one hand, anxiety showed an upward trend, coinciding with the transition to stages of greater academic workload and the onset of clinical demands. This pattern of increasing psychological distress is consistent with national [9] and international [3] literature, which documents that immersion in clinical rotations often triggers and exacerbates anxiety symptoms. In this context, a recent cross-sectional study utilizing the same measurement instrument (Beck Anxiety Inventory) demonstrated that severe anxiety peaks sharply when medical students transition into their clinical years [37]. This evidence underscores the critical need for cognitive buffering resources—such as complex thinking—precisely during these stages.

On the other hand, students’ levels of complex thinking remained statistically stable throughout their entire academic career. Because the interaction test did not show a statistically significant difference in the predictive relationship across academic years, complex thinking functions as a continuous cognitive resource from the beginning of medical training. However, the fact that anxiety symptoms increase significantly while complex thinking levels remain stagnant suggests that students rely on a fixed cognitive baseline to cope with growing academic and clinical demands. Although this baseline provides a consistent protective mechanism, the increasing severity of stressors in advanced years underscores the necessity of actively fostering this competency, rather than assuming it will naturally evolve to meet escalating challenges.

The specific domain analysis revealed a dynamic shift in how students apply cognitive resources. During the first two years, systemic-analysis and creativity scores showed relatively strong inverse correlations with BAI scores. However, during the third and fourth years, as students transition into clinical rotations and direct patient care, problem-solving emerged as the strongest inverse correlate of anxiety. This suggests that while the overall competency level remains stagnant, students adaptively shift their reliance toward the specific cognitive domains most suited to the immediate nature of their academic stressors.

From a cognitive perspective, addressing clinical competence requires shifting from a reductionist approach to an integrative one [18]. By enabling students to understand medical problems as networks of interconnected variables rather than isolated events, complex thinking reduces the sense of cognitive overload, facilitating problem-solving and alleviating anxiety in the face of diagnostic uncertainty.

The stability of complex-thinking scores across the four academic-year groups, despite its inverse predictive relationship with anxiety symptoms, suggests a potential curricular gap. As recent evidence warns, without intentional instructional design, complex thinking does not develop spontaneously or uniformly [38]. In the evaluated context, the results suggest that students may not be developing this competency progressively through the formal curriculum, but rather draw upon it as a pre-existing cognitive resource, developed prior to or independently of the formal curriculum, to cope with the emotional burden of medical training.

To bridge this curricular gap and actively cultivate this competency, medical education must transition toward instructional models centered on integration and active learning. During early foundational years, isolated basic science disciplines can be bridged using interdisciplinary problem-based learning (PBL) modules, which stimulate early systemic analysis. In advanced clinical years, training should transition from algorithmic learning to the management of clinical uncertainty. Within this framework, experiential learning modalities—such as high-fidelity clinical simulations and case-based learning—provide controlled, psychologically safe environments where students can practice multidimensional problem-solving under pressure, followed by structured metacognitive debriefings. Furthermore, performance-based assessments, particularly Objective Structured Clinical Examinations (OSCEs), serve a dual purpose: they act as authentic evaluative tools capable of measuring critical analysis and adaptability, while simultaneously functioning as formative drivers. Ultimately, implementing these insights requires faculty development to shift the focus of routine assessments from rote memorization toward the evaluation of adaptive, complex decision-making.

### Study Limitations

This study had several limitations that must be considered. Methodologically, the cross-sectional design prevented the establishment of definitive causal inferences, making future longitudinal studies necessary to assess the fluctuation of these variables. Furthermore, drawing the sample from a single institution reduced the external validity and the generalizability of the findings to other geographic contexts. Additionally, data collection using self-report instruments may introduce social desirability or response biases.

Conceptually, complex thinking is a multidimensional construct whose theoretical study continues to evolve. Although this research employed a validated and robust instrument for its assessment, the very breadth of this competency suggests that there are still cognitive and attitudinal facets that require further exploration in medical education. Similarly, given the highly dynamic and multifactorial nature of anxiety, our model focused exclusively on this primary predictive relationship, leaving open the need to integrate critical confounding factors in future research. For instance, our sample presented a pronounced gender skew (75.3% female), which is highly relevant given established epidemiological differences in anxiety prevalence between genders. Furthermore, unmeasured covariates such as baseline mental health history, age, and potential attrition bias—where highly anxious students might leave the program before reaching advanced clinical years—could influence the observed anxiety trajectories. Future longitudinal models must adjust for these sociodemographic and clinical covariates to isolate the specific buffering effect of cognitive competencies.

## Conclusions

The present study demonstrates that complex thinking was an inverse predictor of anxiety symptoms across academic-year groups. Although the stratified models showed descriptively higher explained variance in the upper academic-year groups, the interaction analysis did not provide statistical evidence that the strength of this predictive relationship differed across academic years. However, the stagnation of complex thinking levels across academic-year groups suggests a potential curricular gap, as the curriculum may not be actively fostering this competency, leaving students to rely on baseline cognitive resources to manage escalating academic and clinical demands.

These findings suggest that medical schools—particularly those relying on heavily compartmentalized curricula—should evaluate transitioning toward pedagogical models that intentionally integrate systemic and complex reasoning. Furthermore, curricular attention to complex thinking merits further, intervention-based investigation. Future research should prioritize longitudinal designs incorporating contextual covariates (e.g., social support and academic workload) to further elucidate how these cognitive domains evolve and protect students throughout their medical education.

## Data Availability

The datasets generated and/or analyzed during the current study are not publicly available due to institutional privacy and confidentiality restrictions regarding student data, but are available from the corresponding author upon reasonable request.

## Abbreviations

AERA: American Educational Research Association
ANOVA: Analysis of Variance
AVE: Average Variance Extracted
B: Unstandardized regression coefficient
BAI: Beck Anxiety Inventory
CBS: Biological and Health Sciences (Ciencias Biológicas y de la Salud)
CFI: Comparative Fit Index
CI: Confidence Interval
COMPLEX-21: Complex Thinking Scale
CSH: Social Sciences and Humanities (Ciencias Sociales y Humanidades)
df: Degrees of freedom
F: F-statistic
M: Mean
N / n: Total sample size / Subsample size
OSCEs: Objective Structured Clinical Examinations
p: Probability value (p-value)
PBL: Problem-Based Learning
r: Pearson correlation coefficient
R^2^: Coefficient of determination
RMSEA: Root Mean Square Error of Approximation
SD: Standard Deviation
SE: Standard Error
STROBE: Strengthening the Reporting of Observational Studies in Epidemiology
TLI: Tucker-Lewis Index
UNAM: National Autonomous University of Mexico (Universidad Nacional Autónoma de México)
UNESCO: United Nations Educational, Scientific and Cultural Organization
WMA: World Medical Association
α: Cronbach’s alpha
β: Standardized regression coefficient
Ηp^2^: Partial eta squared
